# A call from 40 public health scientists for an end to the continuing humanitarian and environmental catastrophe in Gaza

**DOI:** 10.1186/s12940-024-01097-9

**Published:** 2024-06-28

**Authors:** Leslie London, Andrew Watterson, Donna Mergler, Maria Albin, Federico Andrade-Rivas, Agostino Di Ciaula, Pietro Comba, Fernanda Giannasi, Rima R Habib, Alastair Hay, Jane Hoppin, Peter Infante, Mohamed Jeebhay, Karl Kelsey, Rokho Kim, Richard Lemen, Hester Lipscomb, Elsebeth Lynge, Corrado Magnani, Celeste Monforton, Benoit Nemery, Vera Ngowi, Dennis Nowak, Iman Nuwayhid, Christine Oliver, David Ozonoff, Domyung Paek, Varduhi Petrosyan, Christopher J Portier, Beate Ritz, Linda Rosenstock, Kathleen Ruff, Peter Sly, Morando Soffritti, Colin L. Soskolne, William Suk, Benedetto Terracini, Harri Uolevi Vainio, Paolo Vineis, Roberta White

**Affiliations:** 1 School of Public Health, University of Cape Town, Cape Town, South Africa; 2 Occupational and Environmental Health Research Group, Stirling University, Stirling, Scotland; 3 CINBIOSE, University of Quebec at Montreal, Montréal, Canada; 4 Karolinska Institutet, Solna, Sweden; 5 University of Victoria, Victoria, Canada; 6 University of Bari Medical School, Bari, Italy; 7 Epidemiology Unit, Istituto Superiore di Sanità, Roma, Italy; 8 Civil and Occupational Safety Engineer, São Paulo, Brazil; 9 Faculty of Health Sciences, American University of Beirut, Beirut, Lebanon; 10 Leeds Institute of Cardiovascular and Metabolic Medicine (LICMM), Leeds University, Leeds, UK; 11 Center for Human Health and the Environment, North Carolina State University, Raleigh, USA; 12 OSHA Health Standards, Washington, USA; 13 Brown University, Providence, USA; 14 The Won Jin Occupational Health Foundation and Green Hospital, Seoul, Republic of Korea; 15 Division of Occupational and Environmental Medicine, Duke University Medical Center, Durham, USA; 16 University of Copenhagen, Copenhagen, Denmark; 17 University of Eastern Piedmont at Novara, Novara, Italy; 18 Texas State University, San Marcos, USA; 19 Centre for Environment and Health, KU Leuven, Leuven, Belgium; 20 Institute and Clinic for Occupational, Social and Environmental Medicine, Ludwig Maximilian University (LMU), Munich, Germany; 21 Faculty of Health Sciences, American University of Beirut, Beirut, Lebanon; 22 Dalla Lana School of Public Health, University of Toronto, Toronto, Canada; 23 Boston University School of Public Health, Boston, USA; 24 National Cancer Center, Green Hospital, Goyang-si, Republic of Korea; 25 Turpanjian College of Health Sciences, American University of Armenia, Yerevan, Armenia; 26 CJP Consulting, Thun, Switzerland; 27 UCLA Fielding School of Public Health, University of California Los Angeles, Los Angeles, USA; 28 RightOnCanada, Ottawa, Canada; 29 Children’s Health and Environment Program, Child Health Research Centre, University of Queensland, Brisbane, Australia; 30 Ramazzini Institute, Bologna, Italy; 31 University of Alberta, Edmonton, Canada; 32 Gillings School of Global Public Health, University of North Carolina in Chapel Hill, Chapel Hill, USA; 33 University of Torino, Torino, Italy; 34 Finnish Institute for Occupational Health, Helsinki, Finland; 35 Imperial College, London, UK; 36 United States Assistant Surgeon General (retired), Canton, United States; 37 formerly OSHA Health Standards, Washington, United States; 38 Independent researcher, Dar es Salaam, Tanzania

**Keywords:** War, Gaza, Environmental impacts, Remediation

## Abstract

An under-recognised aspect of the current humanitarian catastrophe in Gaza is the impact of the war on the environment and the associated risks for human health. This commentary contextualises these impacts against the background of human suffering produced by the overwhelming violence associated with the use of military force against the general population of Gaza. In calling for an immediate cessation to the violence, the authors draw attention to the urgent need to rebuild the health care system and restore the physical and human infrastructure that makes a liveable environment possible and promotes human health and well-being, especially for the most vulnerable in the population. Environmental remediation should therefore form one of the most important parts of international efforts to assist reconstruction, through which we hope Palestinians and Israelis will achieve lasting peace, health, and sustainable development, all as part of accepted international human rights obligations.

## Background

As an international group of scientists involved in advancing environmental, occupational, and public health, we call for urgent action to end the current intolerable humanitarian and environmental catastrophe in Gaza. Nowhere in our time has such a confined and captured civilian population as that in Gaza faced so many public health threats from military action in such a short space of time with such a heavy toll on human health and the environment.

Disproportionate force has been employed by Israel resulting in the killing of more than 36,000 people in Gaza, of whom almost one third are confirmed as children. This was in response to an attack by Hamas on Israel on October 7 2023, which resulted in 1200 people killed in Israel and 240 taken hostage [1]. We condemn the use of violence and attacks on civilians and civilian infrastructure, in seemingly clear violation of international humanitarian law. In making this statement, we emphasize that we do not tolerate antisemitism or Islamophobia. And we embrace non-violence as an enduring path towards a just and lasting peace.

## The human toll

By the end of May 2024, around 1.7 million people, representing 75% of Gaza’s population, had been displaced [1]. Nearly half are crammed into the far southern strip, currently under ongoing attack by the Israeli military [2]. More than 80 000 people have been injured and, of people killed for which there is complete information, more than half were women (*n* = 4959) or children under 18 years of age (*n* = 7797)^1^. About 17 000 children are estimated to be orphaned or separated from their parents/families [3–5].

These figures are sourced from the Ministry of Health in Gaza and do not distinguish civilians from fighters. Notwithstanding efforts to dispute the accuracy of these figures [6], independent assessment suggests there is no evidence of inflated mortality reporting from the Gaza Ministry of Health [7]. Indeed, these figures are unquestionably underestimates given that there are many unreported war-related deaths, including of people whose bodies are buried under rubble. Furthermore, they do not include most deaths due to the indirect impacts of war: malnutrition, communicable disease, exacerbations of noncommunicable disease, maternal and infant disorders, and mental and behavioral disorders.

Famine, which UN officials previously warned as being imminent [8] is now established [9] and associated outbreaks of infectious diseases are already evident [10]. Almost the entire Gazan population of 2.2 million people face crisis levels of food insecurity (defined as households unable to consume enough food resulting in high levels of malnutrition or adopting irreversible coping strategies such as selling livelihood-supporting assets to support a limited diet). Amongst them, about 1 million face what is defined as a catastrophic level of food insecurity (defined as people have almost no food and are unable to support their basic needs, despite using all of their coping strategies, with evidence of starvation, death, destitution, and acute malnutrition.) [1]. Levels of acute malnutrition in children under the age of 2 years have reached 31% in some parts of Gaza [1]. Food has been weaponised for military purposes [11] and humanitarian aid subjected to political manipulation through the closure of aid funding to the United Nations Relief and Works Agency (UNRWA) [12].

The situation in Gaza has deteriorated substantially since January 5 2024 when the United Nations noted that “A public health disaster is unfolding. … No food. No water. No school. Nothing but the terrifying sounds of war, day in and day out. Gaza has simply become uninhabitable — while the world watches on” [13]. In May 2024, more than 40% of aid missions in Gaza to areas requiring coordination were reportedly denied or impeded access [1]. As a result, the number of trucks able to deliver food to Gaza is less than 1/5 of what was the case before October 2023, confirming that access to food, water, health care, and other humanitarian aid in the strip is extremely constrained.

The trauma resulting from incessant bombing and unending killing is inflicting mental trauma on the entire population, young and old [14]. More than 1 million children are estimated by the UN Office for Coordination of Humanitarian Affairs as needing mental health and psychosocial support.

Projections made in early February 2024 suggested that if the Israeli bombing continues for another six months (until early August), there could be 58 000 additional deaths [15]. However, this estimate was based on several assumptions, which may turn out to be inaccurate. Nonetheless, even with an immediate ceasefire, the projections suggest that deaths from complications of non-communicable disease and epidemics would be almost three times the number of deaths likely from complications of existing physical trauma, given the absence of a functioning health system in Gaza. Credible sources report a high likelihood of widespread under-reporting of deaths due to acute malnutrition in Gaza [16], confirming the warnings made two months earlier of an imminent public health disaster.

### Infrastructure and environmental impacts

Of Gaza’s 36 hospitals and 93 primary health care centres, the majority are out of service and those hospitals still operating (42%) are only partly functional [1], lacking basic medicines and supplies to treat burn casualties and others seriously injured [17]. At least 493 health workers are estimated to have been killed in Gaza. The fate of the many Palestinian health workers in Gaza held by the Israeli Defence Force (IDF) is unclear with emerging evidence of torture and maltreatment of Gazan health workers in Israeli detention [18–20], including the death of the head of Orthopaedic Surgery at Al-Shifa Hospital, Dr. Adnan Al-Bursh, whilst in Israeli detention [21]. With 86% of elementary and high schools damaged, all universities destroyed, widespread forced displacement, and ongoing violence, no students are attending school in Gaza [1, 22].

Almost 100 university professors have been killed [22], across a range of scientific disciplines, resulting in what Gordon and Turner describe as a process of ‘de-development’ in which ‘whole fields of study’ are decimated for the imminent future [23]. Many of these disciplines represent essential skills needed for the identification and remediation of environmental hazards in Gaza now and in the future. Without a university system, health and environmental professionals will not be able to be trained in Gaza for many years to come, signalling a potentially deep and profound shortfall in future human resources that will be available for health and environmental protection.

More than 230 churches and mosques [22] and half of Gaza’s buildings have been damaged or destroyed, including 60% of its housing stock [24], which amounts to between 144 000 and 175 000 buildings according to analysts from two US Universities [25]. There has been extensive and irreparable damage to museums, archives and libraries [24, 26].

Israeli military attacks have severely reduced access to safe water and sanitation. Israeli forces have bombed water desalination and wastewater treatment plants, leaving two of three water desalination plants only partly functional and have rendered treatment wastewater plants unable to function. This has resulted in approximately 100 000 cubic meters of wastewater being dumped daily into the sea or on land [27], with open sewage in Gazan streets. Access to clean water is minimal [27] and 83% of groundwater wells in Northern Gaza are not operating [24]. Approximately 60% of Water, Sanitation and Hygiene (WASH) facilities have been damaged or destroyed [1]. As a result, water-borne and water-washed diseases are increasing with more than 200 000 acute watery diarrhoea cases reported in February 2024 in Gaza [28]. Outbreaks of hepatitis A have also been recorded [28]. By April, the number of acute diarrhoea cases had risen to 345 000 [29] although the actual number was likely much higher.

The impacts of the war on agriculture have been devastating. By May 2024, 45% of Gaza’s farmland had been razed, 70% of Gaza’s fishing boats destroyed, 60–70% of meat and dairy-producing livestock killed or prematurely slaughtered and 488 wells damaged [1, 24, 30, 31]. There has been an untold amount of damage to animal habitats and ecosystems.

Chemical pollution involving heavy metals and other hazardous materials occurs as a result of weapons used in war [32, 33]. By January 2024, the quantity of bombs released over Gaza was estimated to be over 65 000 tons [34]. Air pollution from detonating explosives, toxic combustion products of fires and particulate matter released from destroyed concrete may have undetermined long-term adverse health impacts, particularly respiratory effects, on surviving civilians [35]. In March 2024, the UN estimated that the war had left 23 million tonnes of rubble and unexploded weapons in Gaza [36], which remain a hazard for the population now and in the future [37]. Early reports suggest emissions in the first two months of the war exceeded the annual carbon footprint of more than 20 of the world’s most climate-vulnerable nations, an impact equivalent to burning more than 150 000 tonnes of coal [38]. Preliminary findings suggest that the carbon costs of post-war reconstruction of the built infrastructure destroyed will total between 46.8 million and 60 million tonnes of carbon dioxide equivalent, emissions higher than the combined annual emissions of over 135 countries, and similar to some High-Income Countries such as Sweden and Portugal [39].

Before the war, Gazans made extensive use of solar power because of the unreliability of electrical power under Israel’s blockade; today, much solar equipment lies in ruin amidst the thousands of buildings destroyed, alongside pulverised building materials containing hazardous materials such as asbestos and other debris [40].

Human Rights Watch reported that White Phosphorus, an incendiary weapon with horrifying potential for human harm and a particularly poisonous pollutant for ecosystems, was used in Gaza [41] in October 2023. This was denied by the Israeli military [42].

If Israeli government plans to flood Hamas’ underground tunnels are executed [43], this will mean that salt water will increase underground water table salinity, reduce the fertility of any farmland, and add to toxic chemical impacts on Gazan soils, as well as reduce access to potable underground water. Scientists have warned this may have ‘a devastating effect on Gaza’s already scarce freshwater supplies and might destabilise buildings’ [44].

The 1998 Statute of the International Criminal Court treats armed attacks that intentionally cause widespread, long-term and severe damage to the environment (referred to as ecocide), disproportionately to any military advantage anticipated, as war crimes. A recent review of the impacts of Israeli military activities on the Palestinian environment since 1948 suggests Israel is in breach of this statute [45]. Moreover, as an occupying power, Israel remains obligated under international human rights law [45, 46] to ensure the right to health of all Palestinians, including the right to a safe environment and the prevention of exposure to “harmful substances … or other detrimental environmental conditions that directly or indirectly impact upon human health” [47].

## Conclusions

The current situation in Gaza is intolerable. As scientists investigating the interface of environmental pollution and human health, we must call attention to the devastating consequences for public health and the lives of future generations resulting from Israeli military action and its impact on the Gazan environment. We echo the call by many groups, including health and human rights organisations within Israel, to stop the attacks on and killing of civilians, to cease attacks on health workers and to protect civilian infrastructure including health facilities, in Gaza [48, 49].

We call on both sides to immediately cease the violence, to release hostages, end imprisonments without fair trial, allow unimpeded access to international humanitarian aid in the whole of Gaza, and open negotiations to ensure a permanent ceasefire that will enable meaningful dialogue between Palestinians and Israelis toward an enduring just and sustainable peace for all in the region.

We urge the global community to support Gaza and the Palestinian people in its post-war recovery and reconstruction, including the rebuilding of the health system, the remediation of environmental pollution arising from the extensive use of weaponry and the implementation of population-wide mental health programmes to address both the current, future and intergenerational effects of trauma. Methods to assess the interplay between trauma, infectious disease, malnutrition and environmental exposures in generating ill-health both in current and future generations and to develop tools to identify those in need of particular types of support, especially children and other vulnerable groups, will be critical for post-war reconstruction.

We believe that health organisations and agencies should join international efforts to assist Palestinians and Israelis in achieving lasting peace, health, and sustainable development. Palestinians were able to build and manage a health system, despite years of blockade on Gaza. International reconstruction commitments in Gaza need to be delivered without any political interference, recognising and respecting the local knowledge, experience and capacity of Palestinians who should lead and implement the rebuilding of the health system and its capacity to address the health and environmental impacts of the war.

## Data Availability

No datasets were generated or analysed during the current study.
